# Supplementary material for: Microbiome Patterns in Matched Bile, Duodenal, Pancreatic Tumor Tissue, Drainage, and Stool Samples: Association with Preoperative Stenting and Postoperative Pancreatic Fistula Development
Source: J Clin Med. 2020 Aug 28;9(9):2785. doi: 10.3390/jcm9092785 (PMC7563524; doi:10.3390/jcm9092785)
Supplement: Supplementary file 1 [file jcm-09-02785-s001.pdf]

Supplementary Materials:

Compared with the bile group, the alpha diversity indices Observed (p-value: 0.0009171; [T-test] statistic: -3.9987), Chao1 (p-value: 0.0012638; [T-test] statistic: -3.8226) and Shannon (p-value: 0.016063; [T-test] statistic: -2.669) in the tumor group were significantly increased. Apart from the alpha indices, beta indices showed, that plots of the PCoA Bray-Curtis dissimilarity (PERMANOVA F-value: 2.6136; R-squared: 0.13325; p-value < 0.012) and PCoA Jensen-Shannon divergence (PERMANOVA F-value: 3.9785; R-squared: 0.18965; p-value < 0.008) as well as NMDS Bray-Curtis (PERMANOVA] F-value: 2.6136; R-squared: 0.13325; p-value < 0.012, NMDS Stress = 0.12267) and NMDS Jensen-Shannon (PERMANOVA F-value: 3.9785; R-squared: 0.18965; p-value < 0.008, NMDS Stress = 0.14698) clearly differentiate between bile and tumor. Then we compared the tumor tissue and the duodenal tissue. Our results showed that the alpha diversity indices Observed (p-value: 0.77995; [T-test] statistic: -0.28365), Chao1 (p-value: 0.84237; [T-test] statistic: -0.20176) and Shannon (p-value: 0.96701; [T-test] statistic: -0.041939) did not differ significantly from each other. Similar to the alpha diversity analysis results, beta diversity analysis through PCoA and NMDS, no significant clustering was observed between duodenal tissue and tumor tissue (PCoA Bray-Curtis: PERMANOVA F-value: 0.16058, R-squared: 0.0088422, p-value < 0.969; PCoA Jensen-Shannon divergence PERMANOVA F-value: 0.17944, R-squared: 0.0098706, p-value < 0.963; NMDS Bray-Curtis: PERMANOVA F-value: 0.17944, R-squared: 0.0098706, p-value < 0.961, NMDS Stress = 0.15361; NMDS Jensen-Shannon divergence PERMANOVA F-value: 0.16058, R-squared: 0.0088422, p-value < 0.967, NMDS Stress = 0.15583). We next compared the composition of the gut microbiome with the tumor microbiome at genus level. The alpha diversity indices Observed (p-value: 6.5503e-07; [T-test] statistic: -7.7837), Chao1 (p-value: 1.8172e-06; [T-test] statistic: -7.0448) and Shannon (p-value: 1.8172e-06; [T-test] statistic: -7.0448) in the gut group were significantly increased. Apart from the alpha indices, beta indices showed, that plots of the PCoA Bray-Curtis dissimilarity (PERMANOVA F-value: 4.7146, R-squared: 0.20756, p-value < 0.001) and PCoA Jensen-Shannon divergence (PERMANOVA F-value: 7.3332, R-squared: 0.28947, p-value < 0.001) as well as NMDS Bray-Curtis (PERMANOVA F-value: 4.7146, R-squared: 0.20756, p-value < 0.001, NMDS Stress = 0.11408) and NMDS Jensen-Shannon (PERMANOVA F-value: 7.3332, R-squared: 0.28947, p-value < 0.001, NMDS, Stress = 0.15228) clearly differentiate between gut and tumor.
